# Multi-species and multi-tissue methylation clocks for age estimation in toothed whales and dolphins

**DOI:** 10.1038/s42003-021-02179-x

**Published:** 2021-05-31

**Authors:** Todd R. Robeck, Zhe Fei, Ake T. Lu, Amin Haghani, Eve Jourdain, Joseph A. Zoller, Caesar Z. Li, Karen J. Steinman, Stacy DiRocco, Todd Schmitt, Steve Osborn, Bill Van Bonn, Etsuko Katsumata, June Mergl, Javier Almunia, Magdalena Rodriguez, Martin Haulena, Christopher Dold, Steve Horvath

**Affiliations:** 1 Zoological Operations, SeaWorld Parks and Entertainment, Orlando, FL USA; 2 Department of Biostatistics, Fielding School of Public Health, University of California, Los Angeles, Los Angeles, CA USA; 3 Department of Human Genetics, David Geffen School of Medicine, University of California, Los Angeles, Los Angeles, CA USA; 4 Norwegian Orca Survey, Andenes, Norway; 5 Species Preservation Laboratory, SeaWorld San Diego, San Diego, CA USA; 6 SeaWorld Orlando, Orlando, FL USA; 7 SeaWorld San Diego, San Diego, CA USA; 8 SeaWorld San Antonio, San Antonio, TX USA; 9 John G. Shedd Aquarium, Chicago, IL USA; 10 Kamogawa Sea World, Kamogawa, Chiba Japan; 11 Marineland of Canada, Niagara Falls, ON Canada; 12 Loro Parque Fundación, SA, Avenida Loro Parque, Puerto de la Cruz, Santa Cruz de Tenerife Spain; 13 Miami Seaquarium, Miami, FL USA; 14 Vancouver Aquarium, Vancouver, Canada

**Keywords:** DNA methylation, Conservation biology

## Abstract

The development of a precise blood or skin tissue DNA Epigenetic Aging Clock for Odontocete (OEAC) would solve current age estimation inaccuracies for wild odontocetes. Therefore, we determined genome-wide DNA methylation profiles using a custom array (HorvathMammalMethyl40) across skin and blood samples (n = 446) from known age animals representing nine odontocete species within 4 phylogenetic families to identify age associated CG dinucleotides (CpGs). The top CpGs were used to create a cross-validated OEAC clock which was highly correlated for individuals (r = 0.94) and for unique species (median r = 0.93). Finally, we applied the OEAC for estimating the age and sex of 22 wild Norwegian killer whales. DNA methylation patterns of age associated CpGs are highly conserved across odontocetes. These similarities allowed us to develop an odontocete epigenetic aging clock (OEAC) which can be used for species conservation efforts by provide a mechanism for estimating the age of free ranging odontocetes from either blood or skin samples.

## Abstract (web summary)

Todd Robeck and Zhe Fei et al. use DNA methylation profiling to develop an epigenetic clock to measure age in odontocete species, including toothed whales and dolphins. The clocks were highly accurate and represent a valuable tool to help in wildlife conservation efforts.

## Introduction

Accurate age estimation of wild odontocetes (toothed whales) is an important component of any population health assessment and is critical for the development of management plans designed to help wild populations in need. Current age estimations typically rely on a combination of techniques including animal length, long-term capture and recapture of animals using photo identification, and population biology (eg., age at sexual maturity, calving intervals, age at reproductive senescence) that are selectively applied toward individuals. In general, age estimators based on allometry or life history become less accurate once physical maturity has been reached and surpassed^1–4^.

Although long-term photo identification studies have produced the most robust and accurate data concerning life history of a few populations of odontocetes, these studies face limitations^2–5^, for example, inaccuracies can arise when attempting to use population level biological statistics to define ages of individual animals that were mature prior to the onset of these studies^3,6^. Within population level management plans, erroneous age assignment in a few animals does not have adverse consequences when the error is averaged out. However, wrong age estimation in older animals can lead to misleading extrapolations about the normal life expectancies and the erroneous identification of an abnormally long post-reproductive life span for select species such as killer whale or beluga^6–8^. Therefore, verifying the prevalence and length that females typically live beyond fertility is critical for discussions surrounding behavior (eg., learning and cultural transmission) and population assessments^6–8^.

Although long-term photo identification programs are often used for estimating life history parameters, their high economic costs and long-term time commitments render them less useful for time-sensitive management decisions for species that need immediate protection^9^.

Ages of odontocetes can be estimated based on tooth sectioning and then counting of tooth growth layer groups (GLGs^10^). Although GLGs represent the current gold standard for determining the age of odontocetes, this method is not without controversy because pulling a tooth is impractical in most odontocetes larger than bottlenose dolphins and it may be considered too invasive during standard health assessments. Finally, accuracy and debate exist concerning the ability to count growth layers as teeth wear differentially based on feeding strategies or simply with age^11–18^.

In addition to potential inaccuracies with age, for one family of odontocete, the Monodontidae (beluga and narwhal), a consensus has not been reached concerning the rate of deposition of GLGs of either one or two GLGs per year^18–25^. Moreover, and irrespective of methodological variations, a GLG deposition of one per year consistently overestimates important biologic milestones (age at sexual maturation, age at reproductive senescence) and ages of documented, known aged animals^23,26–28^.

Multiple other aging methods have been evaluated for use with cetaceans that appear to be of value include eye lens aspartic acid racemization^29–32^, fatty acid composition^33–35^, radiocarbon 14 dating from fallout^25^, and telomere length^36^. However, all methods must be calibrated against some age estimate of the species in question, most often by using GLG counts, and as suggested for aspartic acid validation^32^, validation of all tests would be improved using known age animals^35^. In addition to a need for standardization against some known age, many of these techniques can be affected by environmental conditions (particularly fatty acids composition) and often display wide ranges of age estimations for animals of similar chronological age^1,35^. Finally, telomere length change over time appears to have too much variation among individuals to be used as an accurate age predictor, and within cetaceans, its accuracy has been demonstrated to be negatively affected by a high inherent individual variability among cetaceans, high amount of interstitial telemetric sequences, and the influence of paternal age^1,35,37,38^.

In an attempt to improve upon age estimations of wild cetaceans, two DNA methylation or epigenetic aging clocks directed at individual species have been published for the humpback whale^39^, Antarctic minke whale^40^, and the bottlenose dolphin^1^. DNA methylation is a well-studied epigenetic modification which refers to the transfer of a methyl (CH3) group from S-adenosyl methionine (SAM) to the fifth position of cytosine nucleotides, forming 5-methylcytosine (5mC)^41^. These epigenetic changes can result in the modulation of gene activity without any changes in the genomic sequence^41^.

It has been observed that the degree of DNA methylation is influenced by age^42,43^. Along with the development of an array-based technology that permits quantification of methylation levels of specific CpG positions on the human genome came the insight that one can combine age-related methylation changes of multiple DNA loci to develop a highly accurate age-estimator (epigenetic clock) for all human tissues [reviewed in refs. ^44,45^]. For example, the human pan-tissue clock combines the weighted average of DNA methylation levels of 353 CpGs into an age estimate that is referred to as DNA methylation age (DNAm age) or epigenetic age^46^. Epigenetic clocks are regarded as the most accurate molecular estimators of chronological age^44,47^. The hope of extending the benefits of these epigenetic clocks to other animals was initially encouraged by the direct applicability of the human pan-tissue clock on chimpanzee DNA methylation profiles. This compatibility, however, could not be extended to other animals because of evolutionary genome sequence divergence^46^. Hence, it is necessary to develop de novo epigenetic clocks specific to animals of interest as, for example, what has been accomplished for mice^48–53^. However, as described for other methods for age determination in cetaceans, development of these clocks using known age animals is required for correct calibration.

Our objectives were to address this issue within odontocetes and to determine if a DNAm epigenetic aging clock that relies on highly conserved stretches of DNA across taxa using known age animals could be developed. Here, we present DNA methylation-based age estimators (epigenetic clocks) for odontocetes that work for skin and/or blood samples. In general, these molecular age estimators for odontocetes could find two major areas of application: (1) animal conservation efforts; and (2) comparative studies in the biology of aging.

## Results

We obtained DNA methylation profiles from blood and skin (*n* = 446) samples from 267 animals across nine species of odontocetes ranging in ages from 0 to 58 years of age (Table 1). For these species we had either blood or skin samples or both. An unsupervised hierarchical analysis clustered the methylation profiles primarily by tissue type (blood, skin, other tissues) and, to a lesser extent, by species was used to identify conserved CpG sites to be screened for potential use in the epigenetic clock development (Supplementary Fig. 1).

**Table 1:** Demographic information of the odontocete species for which DNA methylation data were available.

Species	Common name	Origin	N^a^	No. animals	No. female	Mean age	Median age	Minimum age	Maximum age
*Cephalorhynchus commersonii*	Commerson’s dolphin	Zoo born	6 (3)	3	1	22.4	21.3	18.6	27.5
		Wild	1 (1)	1	0	36.1	36.1	35.1	37.0
*Delphinapterus leucas*	Beluga	Zoo born	65 (48)	42	20	9.8	8.0	0.15	27.3
		Wild	35 (17)	19	11	27.9	32.5	1.2	49.2
*Delphinus delphis*	Common dolphin	Wild	6 (3)	3	2	14.8	14.8	2.6	26.2
*Globicephala macrorhynchus*	Short-finned pilot whale	Wild	15 (9)	7	3	16.1	8.8	6.5	55.3
*Lagenorhynchus obliquidens*	Pacific white-sided dolphin	Zoo born	26 (13)	14	7	9.0	7.4	0.4	19.8
		Wild	8 (4)	4	4	34.0	31.9	31.9	40.4
*Orcinus orca*	Killer whale	Zoo born	56 (29)	30	17	15.7	13.9	0.26	30.9
		Wild	32 (7)	26^b^	19	22.9	21.0	0	54.6
*Phocoena phocoena*	Harbor porpoise	Wild	2 (1)	1	1	17	17	17	17
*Steno bredanensis*	Rough-tooth dolphin	Wild	12(6)	6	4	13.6	14.6	4.6	24.0
*Tursiops truncatus*	Bottlenose dolphin	Zoo born	160 (123)	120	81	16.5	14.5	0.57	40.7
		Wild	21(17)	17	11	35.0	36.4	3.9	58.0

^a^Total number of samples used for clock development, number of blood samples in parenthesis.

^b^Total number of animals includes 19 samples from wild Norwegian killer whales of unconfirmed sex (unless mature) that were used in clock development.

### Epigenetic clocks

**Fig. 1:**
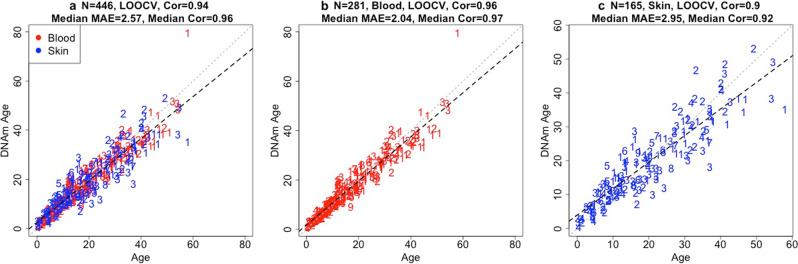
Cross-validation study of the epigenetic clock for odontocetes. Each dot corresponds to a tissue sample from odontocetes. Dots are colored by tissue type (red = blood, blue = skin) and labeled by species: 1 = bottlenose dolphin (*Tursiops truncatus*); 2 = beluga (*Delphinapterus leucas*); 3 = killer whale (*Orcinus orca*); 4 = Pacific white-sided dolphin (*Lagenorhynchus obliquidens*); 5 = short-finned pilot whales (*Globicephala macrorhynchus*); 6 = rough-toothed dolphin (*Steno bredanensis*); 7 = Commerson’s dolphin (*Cephalorhynchus commersonii*); 8 = common dolphin (*Delphinus delphis*); 9 = harbor porpoise (*Phocoena phocoena*). **a**–**c** represent cross validations of different tissue strata, with blood and skin combined (**a**), blood only (**b**), and skin only (**c**). LOOCV corresponds to leave-one-sample out cross validation (LOOCV) estimates. Each panel depicts a linear regression line (black dashed line), a diagonal line (y = x), the sample size (*N*), Pearson correlation (Cor) across all samples, median age correlation across species, and median value of the median absolute error (MAE) across species.

From the methylation data, we generated three primary odontocete epigenetic clocks that differ with regards to applicability to different tissue types: blood and skin clock; blood clock; and skin clock. Our three odontocete clocks used all available species but were trained on different combinations of tissues: both blood and skin (clock based on 142 CpGs); blood only (clock based on 58 CpG); or skin only (clock based 79 CpGs, Supplementary Data File 1). To arrive at unbiased estimates of predictive accuracy, we carried out two cross-validation schemes (Leave One Sample Out Cross Validation [LOOCV] and Leave One Species Out Cross Validation [LOSOCV]) to estimate two measures: the age correlation r (defined as Pearson moment correlation between the age estimate, DNAm age, and chronological age), as well as the median absolute error (MAE, in units of years). The three odontocete clocks were highly accurate based on LOOCV (blood and skin clock: *r* = 0.94; MAE = 2.57 years [Fig. 1a]; blood clock: *r* = 0.96; MAE = 2.04 years [Fig. 1b]; skin clock: *r* = 0.9; MAE = 2.95 years [Fig. 1c]). In addition, the high accuracy according to LOSOCV results from blood and skin clock (median *r* = 0.94, median MAE = 4.21 years [Supplementary Fig. 2a]); blood clock (median *r* = 0.94, median MAE = 3.34 years [Supplementary Fig. 2b]) and skin clock (median *r* = 0.9, median MAE = 7.76 years [Supplementary Fig. 2c]) indicate that these clocks are applicable to any odontocete species that were not part of the original training set.

**Fig. 2:**
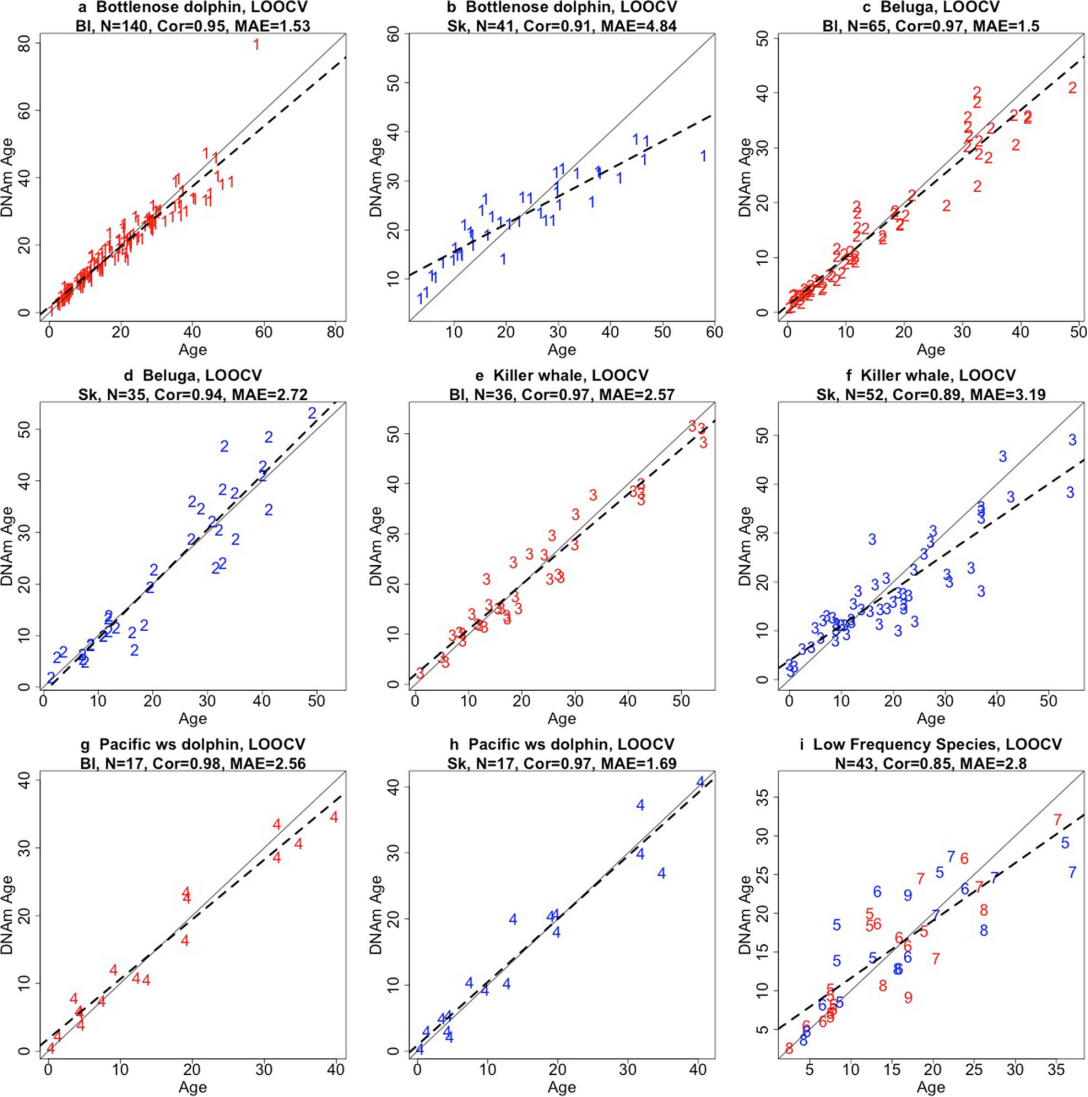
Cross-validation (LOOCV) study of the odontocete blood + skin clock (OEAC) applied to skin and blood samples collected from individual species. Each panel reports leave-one-out (LOOCV) estimates of the odontocete clocks applied to blood and skin samples from each species: bottlenose dolphin (**a**, **b**); beluga (**c**, **d**); killer whale (**e**, **f**); Pacific white-sided (ws) dolphin (**g**, **h**); and low frequency species (**i**). Dots are colored by tissue type (red = blood, blue = skin). Numbers denote different species: 1 =  bottlenose dolphin (*Tursiops truncatus*); 2 = beluga (*Delphinapterus leucas*); 3 = killer whale (*Orcinus orca*); 4 = Pacific white-sided dolphin (*Lagenorhynchus obliquidens*); 5 = short-finned pilot whales (*Globicephala macrorhynchus*); 6 = rough-toothed dolphin (*Steno bredanensis*); 7 = Commerson’s dolphin (*Cephalorhynchus commersonii*); 8 = common dolphin (*Delphinus delphis*); and 9 = harbor porpoise (*Phocoena phocoena*). Each panel depicts a linear regression line (black dashed line), a diagonal line (y = x), the sample size (*N*), Pearson correlation (Cor), and median absolute error (MAE) across all samples.

The application of the Odontocete Epigenetic Aging Clock (OEAC) clock toward samples from individuals within each species included in the training set indicated unbiased (LOOCV) age estimations of high accuracy for each species. Estimates from blood samples had correlations that ranged from *r* = 0.95 (MAE = 1.5 years) for bottlenose dolphins (Fig. 2a) to *r* = 0.98 (MAE = 2.6 years) for Pacific white-sided dolphin (Fig. 2g). Correlations for skin samples ranged from *r* = 0.89 (MAE 3.2) for killer whales (Fig. 2f) to *r* = 0.97 (MAE = 1.7 years) for the Pacific white-sided dolphin (Fig. 2h). Estimates from low frequency species, species with ≤15 samples in total (Table 1), were combined for illustration purposes resulting in a correlation of *r* = 0.85 (MAE = 2.8 years, Fig. 2i). Across the predictions, whereby species had both blood and skin samples, blood samples had a median 3.2% improvement in accuracy and a 1.31 year decrease in MAE.

### Age prediction versus GLG layers in beluga teeth

Ages of two beluga were predicted from blood samples of known age (KA) or estimated age (EA, allometric estimation of juvenile at collection) within this study. GLG layers in teeth from these animals had previously GLG been counted^22^. The known age or EA of the beluga, and unbiased DNAm predicted age and GLG counts were as follows: Animal 1, EA = 29.25 y, DNAm age = 32.4 y and 33.0 (from two samples), GLGs = 55–60+; Animal 2: KA = 21.3 y, DNAm age = 17.8 y, GLGs = 40–42.

### Epigenetic estimator of sex, tissue, and species

We built random forest predictors for three categorical variables: species; tissue type (skin versus blood); and sex across the eight cetacean species. In other words, we ignored species when fitting the random forest predictors. The random forest predictor for species led to nearly perfect accuracy with the out-of-bag (OOB) estimate of the error at 1.5%. The error arose from species with low tissue counts (*N* = 2 for harbor porpoise, *N* = 4 for common dolphin). The random forest predictor of blood versus skin was perfectly accurate (OOB error = 0%).

The random forest predictor of sex-associated differences in DNAm across the various odontocete species led to an OOB error of 0.5 percent, i.e., it misclassified two animals. We identified four CpGs that are highly differentially methylated across sex (Supplementary Fig. 3). Three CpG probes (“cg00878023”,“cg03341064”,“cg15281901”) assume lower methylation values in male than in female odontocetes (*p* < E-59). Since these three CpGs map to the X chromosome in humans, it is likely that these CpGs map to X chromosome in odontocetes as well but the final confirmation awaits an improved genome assemble in these species. In general, X chromosomal cytosines are hypermethylated in females, which accompanies X-inactivation. Another probe, “cg15451847”, took higher values in males than in females (p = 2.3E-61). This probe is a Y chromosome CpG in human.

### Epigenome-Wide Association Studies of chronological age-related CpGs in Odontoceti samples

In total, 30,467 probes from the HorvathMammalMethylChip40 could be mapped to the killer whale genome assembly (GCF_000331955.2_Oorc_1.1). These 30,467 are proximal to 6209 genes in the killer whale genome. By design, CpG probes on the methylation array are located in DNA stretches that exhibit high inter-species conservation. Therefore, most of these CpGs should also map to other Odontoceti. For example, ~50% of probes aligned proximate to identical genes between odontocete species (Supplementary Fig. 4). The probes that were mapped to different genes were mainly located in intergenic regions that are distant from the coding sequences (47%).

**Fig. 3:**
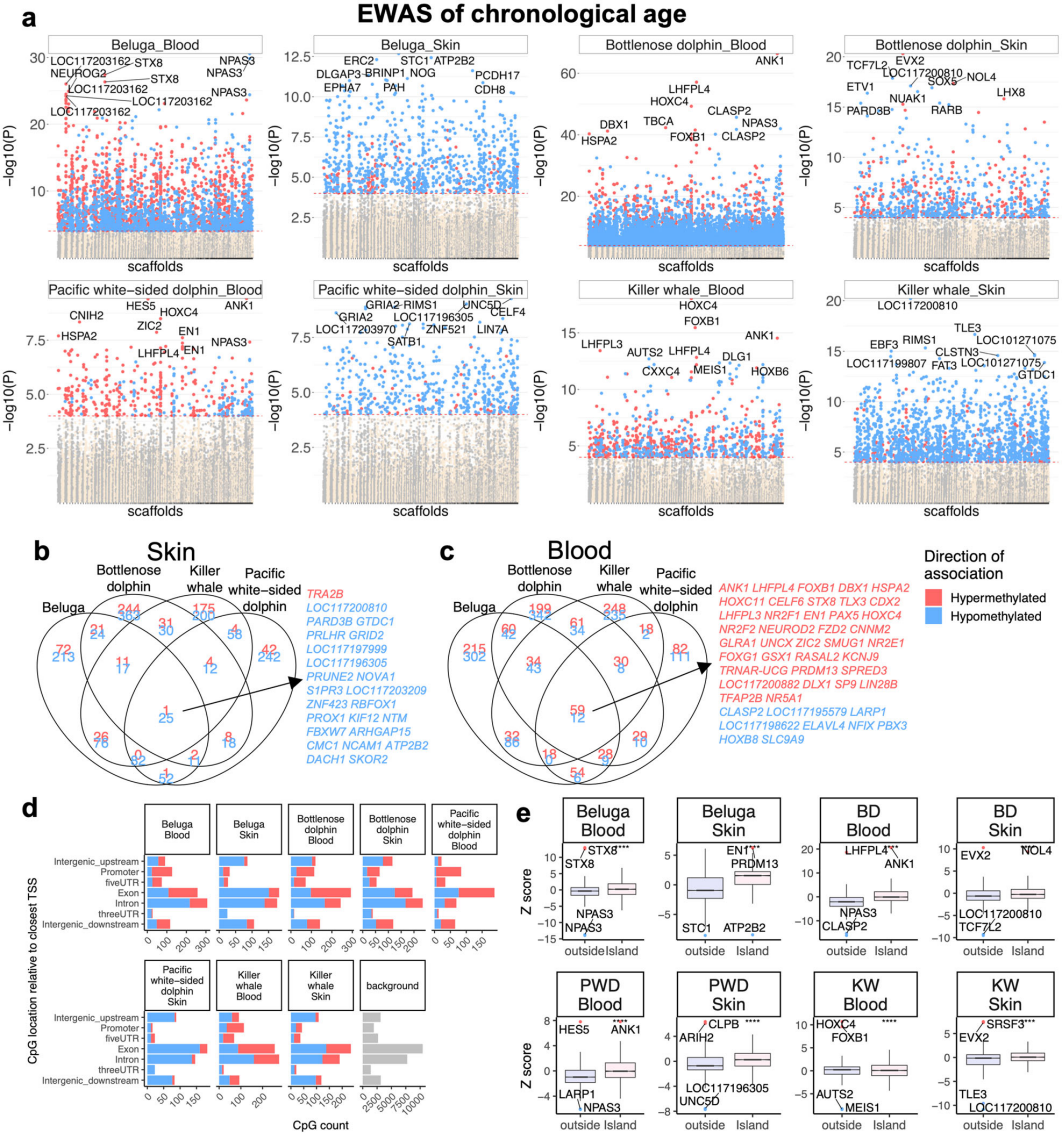
Epigenome-wide association (EWAS) of chronological age in skin and blood of four Odontoceti species. **a** Manhattan plots of the EWAS of chronological age. The coordinates are estimated based on the alignment of Mammalian array probes to killer whale (GCF_000331955.2_Oorc_1.1) genome assembly. The direction of associations with *P* < 0.0001 (red dotted line) is highlighted by red (hypermethylated) and blue (hypomethylated) colors. Top 10 CpGs were labeled by the neighboring genes. Venn diagrams representing the overlap of aging-associated CpGs in skin (**b**) and blood (**c**) of these species. The conserved DNAm aging loci in blood and skin samples were marked by neighboring gene symbol. This analysis was limited to top CpGs that were selected at *P* < 0.0001 and further filtering based on *z* score of association with chronological age for up to 500 in a positive or negative direction. The number of selected CpGs: beluga blood, 1000; beluga skin, 639; bottlenose dolphin blood, 1000; bottlenose dolphin skin, 68; Pacific white-sided dolphin blood, 490; Pacific white-sided dolphin skin, 564; killer whale blood, 935; killer whale skin, 665. **d** Location of top CpGs in each species relative to the closest transcriptional start site. **e** Box plot represents 25th and 75th percent quartiles, the line represents the median, and whiskers are 90% of aging association stratified by CpG island status in killer whale genome. *p < 0.05, **p < 0.01, ***p < 0.001, ****p < 0.0001.

Epigenome-wide association studies (EWAS) of chronological age revealed a tissue-specific DNAm change in the Odontoceti (Fig. 3a). In general, blood and skin samples had distinct DNAm aging patterns in all four odontocete species (beluga, bottlenose dolphin, killer whale, and Pacific white-sided dolphin) used for this analysis. An upset plot, which can be interpreted as a generalization of Venn diagrams of overlap, revealed several CpGs whose strong age correlations can be observed in at least four odontocete species, particularly in blood samples (Fig. 3b). Aging-associated CpGs in odontocetes were distributed in all genic and intergenic regions that can be defined relative to transcriptional start sites (Fig. 3c). A consistent pattern in blood samples was an increase of methylation in gene promoters. However, the skin samples did not follow this pattern, which may reflect lower sample sizes for the skin samples in some species examined (blood: range 17–140 samples per species; skin: range 17–52 samples per species; Fig. 3d). Thus, the observed differential methylation pattern should be re-examined in future studies.

Aging mediated hypermethylation in promoters paralleled a higher positive association of CpG islands with age than non-island CpGs in beluga (particularly skin), BD, and PWD blood and skin samples (1-30% higher median *z* score, Fig. 3e). In contrast to these three species, CpG islands had a small median age association (1–2%) difference with non-island CpGs in KW samples.

**Fig. 4:**
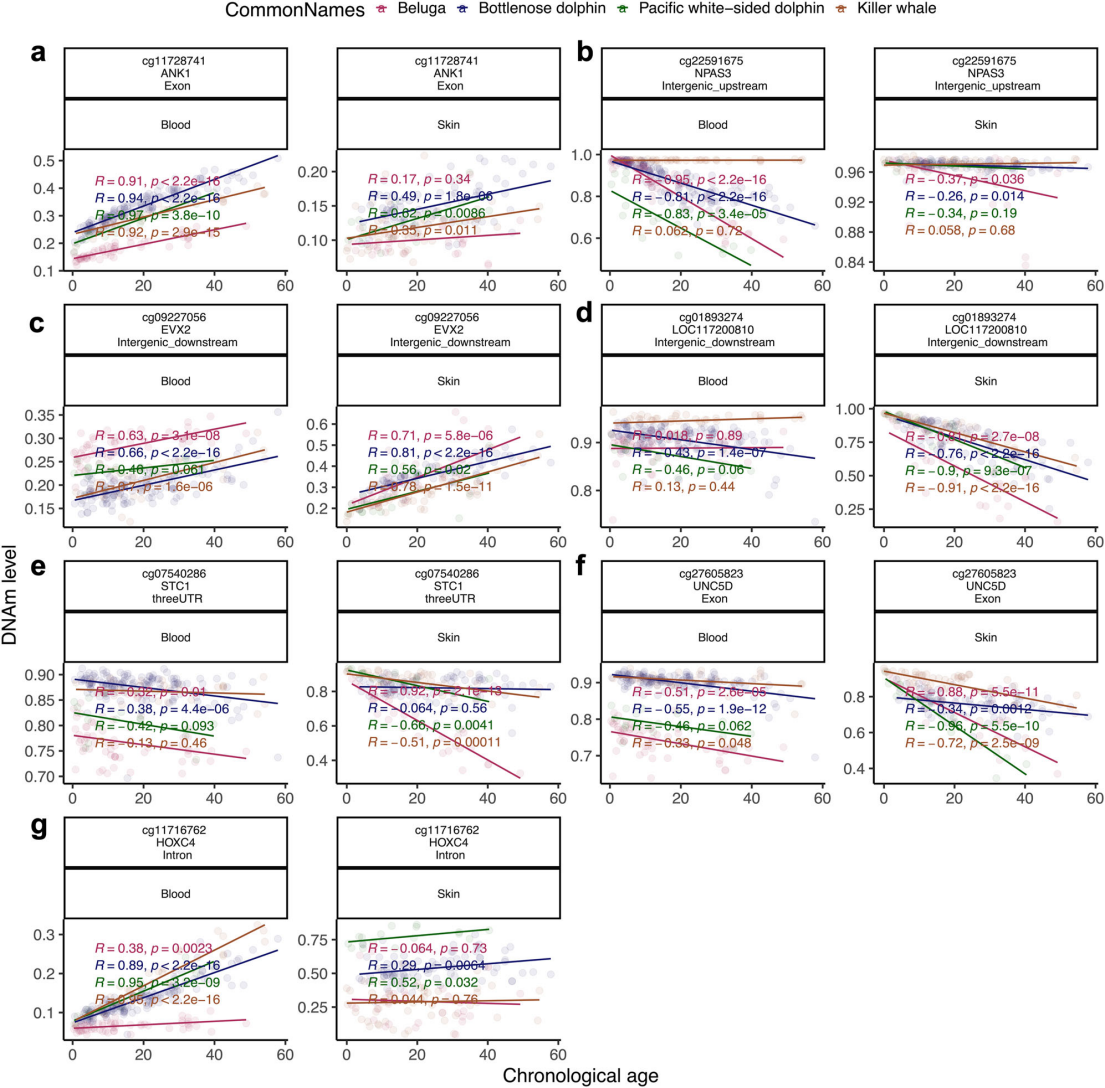
DNAm aging in top CpGs from EWAS of chronological age in blood and skin of odontocetes. Top CpGs proximate to genes: **a** *ANK1* exon, which was the top region in the blood of bottlenose (*z* = 20.6) and Pacific white-sided (*z* = 7.8) dolphins; **b** *NPAS3* upstream from beluga blood (*z* = −14) EWAS; **c** EVX2 downstream from bottlenose dolphin skin (*z* = 10.3); **d** *LOC117200810* downstream from killer whale skin (*z* = −10.9) EWAS; **e** *STC1* 3′UTR from beluga skin EWAS (*z* = −8.5); **f** *UNC5D* exon from Pacific white-sided dolphin skin (*z* = −7.7); **g** *HOXC4* intron from killer whale blood (*z* = 10.7). The coordinates are estimated based on the alignment of Mammalian array probes to killer whale (GCF_000331955.2_Oorc_1.1) genome assembly.

To capture the top affected loci in all species, DNAm was studied at a nominal *P* < 0.001. The top DNAm changes and the proximate gene in each species and tissue are as follows (Fig. 4a–g): beluga, *NPAS3* upstream (correlation test Z statistic *z* = −14) in blood (Fig. 4b) and *STC1* 3′UTR (*z* = −8.3) in skin (Fig. 4e); bottlenose dolphin (BD), *ANK1* exon (*z* = 20) in blood (Fig. 4a) and *EVX2* downstream (*z* = 10.3) in skin (Fig. 4c); Pacific white-sided dolphin (PWD), *ANK1* exon (*z* = 7.8) in blood (Fig. 4a) and *UNC5D* exon (*z* = −7.7) in skin (Fig. 4f); and killer whale (KW), *HOXC4* intron (*z* = 10.7) in blood (Fig. 4g) and *LOC117200810* downstream (*z* = −9) in skin (Fig. 4d). Interestingly, the changes in almost all these top CpGs had similar patterns in the blood and skin of these four odontocete species. However, some exceptions merit further discussion. For example, although the *NPAS3* upstream region was hypomethylated with age in blood of beluga, PWD, and BD, it did not show any change in killer whales (Fig. 4b). Moreover, this CpG was only changed in the beluga skin. Another example is *HOXC4* intron, which had a lower rate of age mediated hypermethylation in beluga blood than others (Fig. 4g). This suggests while DNAm aging has several similar aspects in odontocete, some unique changes potentially contribute to species level phenotypes.

Gene level enrichment analysis of the significant CpGs highlighted changes in development, the nervous system, and metabolism (Supplementary Fig. 5). Further, we find enrichment of *H3K27Me3* marks and polycomb protein EED for genes proximate to CpGs that exhibit positive age correlations. EED is a member of the multimeric Polycomb family protein complex that maintains the transcriptional repressive states of genes. These proteins also regulate *H3K27Me3* marks, DNA damage, and senescence states of the cells during aging^54^.

### Application of OEAC to Norwegian killer whales of unconfirmed age

Of the 45 skin samples from free-ranging Norwegian killer whales, five could not be analyzed because an inadequate amount of DNA. Nineteen were used for clock development and the OEAC predicted ages for the remaining 21 animals. In addition to age, we were able to predict sex, (see next section) and produce a population demographic chart for all free-ranging killer whales used within this study (Supplementary Fig. 6). The overall sex ratio was significantly (*P* = 0.005) skewed beyond an even sex ration (50/50) toward males (31 Male /13 female). This skew, although not significant was also observed in animals <10 years of age (5/2). Overall, the population percentages were as follows: Juveniles 30.0%; Adult females (>9 y) 27.5%; Adult males (>13 y to <41 y) 47.5%; Post-reproductive females (>40 y) none; Aged (>40 y) 5%. The lack of samples collected from females over age 40 is less (*P* = 0.04) than the expected number of 3.08 (7.7%) as compared to wild northern resident killer whale populations of British Columbia^3^.

## Discussion

This study describes the construction of a accurate (*r* = 0.94 LOOCV) odontocete DNAm epigenetic clock that was developed using blood and skin samples from eight species within four families of odontocetes. In addition, the LOSOCV cross-validation analysis indicated that the chronological age of an animal from any species of odontocete can be predicted with a high degree of accuracy (median correlation across species *r* = 0.94) using blood or skin samples. The custom methylation array that profiles cytosines in highly conserved stretches of DNA allowed us to analyze DNA methylation profiles from several different odontocetes. By using the same DNA methylation measurement platform, it was straightforward to create OEACs that apply to skin or blood or both.

Similar to the only other epigenetic clock produced for an odontocete, the bottlenose dolphin (BEAT^1^), our data stand out because they were mainly comprised of animals whose ages were known precisely (often the birthday was known) or whose age could be estimated with high accuracy. The use of known age animals for the development of the OEAC allowed us to circumvent the ambiguity of other cetacean aging methods whose limitations have been discussed in the literature^4,6,23^. Resolution of these controversies could easily be accomplished through the application of our clock. For example, blood and skin samples from wild beluga that are currently aged using growth layer groups (GLGs) are often collected during heath assessment programs or post-mortem after subsistence hunting^55–57^ and could be used within the OEAC system for age determination. The ages predicted by our clocks could then be compared to GLG counts to statistically determine the number of GLGs deposited per year. Obviously, if predicted age is ~50% of the total numbers of GLG counts per animal, then the evidence would support our limited findings herein from known age animals that ~two GLGs are deposited per year in beluga^22,23^. Future efforts should be directed at providing an answer to this important question.

Although our epigenetic clocks were primarily developed on the basis of zoological specimens, we are confident that these clocks would also apply to wild odontocetes. Evidence from human epidemiological studies suggests that any differences between these two populations of animals, in terms of age acceleration due to lifestyle or anthropogenic stress, would have a relatively weak effect on DNA methylation aging^44,58^. In addition, the most pronounced age acceleration effects are detected in the tissues from the organ that is primarily affected by the exogenous insult (e.g., liver in environmental toxic insults). Finally, the Horvath clock has been calibrated for and demonstrated to be highly correlated with chronological age and in its current iteration has had limited application toward the detection of sites vulnerable to age acceleration^46,58,59^. Therefore, it is highly unlikely that in its current configuration, or CpG site selection, differences in aging-associated methylation changes would exist between free-ranging and zoo-based animals. However, genetic differences or differences in DNA sample processing could lead to a systematic offset in the age estimates. To estimate such an offset, including more known age animals in future test data will be useful.

It is widely acceptable that methylation rates are highly conserved within blood, and blood has been the tissue of choice for most epigenetic clock development in both human and animals. For species within our study, whereby we could directly compare the OEAC predictions against blood or skin samples, we consistently saw improved prediction and reduced variability when using blood versus skin samples. In addition, our species cross correlated (LOSOCV) odontocete blood clock (*r* = 0.88) was also improved compared to our skin clock (*r* = 0.67). Although these lower correlations for clocks that rely on skin samples could simply be the results of reduced sample sizes, they may also be indicative of more variability in DNAm within the tissue stratum of skin samples. Because the majority our samples were collected by multiple veterinarians across multiple institutions, differences in skin scraping techniques and site locations may have resulted in variations in the sample collected – dermal, epidermal, or both. In humans, evidence indicates differential methylation rates between tissues (this study^60,61^), and uniformity in sample site collection, specifically dermal versus epidermal^62^, is considered important for reducing intra-individual variances^62^. Future efforts could be made to determine if sample location, depth and presence and/or absence of skin lesions affect methylation rates in cetacean skin and, furthermore, standardized skin collection protocols could be developed.

On the surface, our OEAC appears to have demonstrated improved accuracy (*r* = 0.91, MAE = 4.8 years) at predicting ages of bottlenose dolphin with skin samples when compared to the results of the recently published bottlenose dolphin epigenic aging clock (BEAT, *R*^2^ = 0.74, rms = 5.1 years, 1) which was developed using only skin samples. Although it is difficult to compare the accuracy of these different clocks unless they are tested on the same samples, obvious differences between the two clocks are in the total numbers of CpGs used and the statistical analysis for clock development (multiple linear regression versus LASSO regression). For BEAT development, Beal et al.^1^, have determined that two of 17 CpG age-related sites on two genes (*TET2*: CpG site 2, and *GRIA2*: CpG site 5) provided the best regression model for BEAT development. In contrast, we directly modeled 38k CpGs and identified (using LASSO regression) 142 CpGs for OEAC development across 9 species that were significantly correlated with chronological age. Therefore, our apparent increased accuracy compared to Beal et al.^1^ is most likely due to the increased CpG sites and skin samples across multiple odontocetes we used for clock development. It has been documented that sample size and total number of methylation sites identified affect clock accuracy^59^.

In addition to age estimations in bottlenose dolphins, Beal et al^1^. suggest that the BEAT tool is appropriate for use with other small odontocetes without providing supporting evidence. Using the LOSOCV cross validation, we provided evidence that the OEAC can accurately (median *r* = 0.94, median MAE = 4.21 years) provide age estimations against any unknown odontocete species, and thus, the OEAC is a validated tool for predicting the age of any odontocete species. Future use of the OEAC to identify the ages of other species not used in clock development will help provide evidence for or against our supposition.

Besides epigenetic clock development, the mammalian array is a unique reproducible tool for a direct genome-wide comparison of DNAm changes in cetaceans and other mammalian species. Our EWAS identified DNAm aging proximate to 1064 genes with some tissue-specificity in odontocete species. Functional analysis of the genes with DNAm aging highlighted processes related to development, nervous system, survival, and even some age-related diseases such as cancer. The top seven age-related genes further confirm that these changes are related toward aging biology. For example, *ANK1* is differentially methylated during Alzheimer’s disease^63^ and have genetic variants associated with a risk of type 2 diabetes^64^. *NPAS3* and *MEF2C* are involved in neurodevelopment^65,66^. The other three genes (*STC1*, *UNC5A*, and *HOXC4*) are implicated in several cancer types such as colorectal cancer^67^ and hepatocellular carcinoma^68^. DNAm aging in *STC1* specifically correlates with methylation changes associated with the inflammatory processes^69,70^. All these pieces of evidence suggest a resemblance in the age-related biological pathways between humans and odontocete. The identified genes merit additional mechanistic experiments to resolve differential aging phenotypes between humans, other mammals, and these cetacean species.

For comparison, the BEAT clock^1^ identified two CpG sites on two genes, *TET2* (CpG site 2) and *GRIA2* (CpG site 5) that accounted for 78% of the age-related variation in % DNA methylation, respectively. These genes were also found to correlate with age in humpback whales^39^. Surprisingly, although we had several probes that mapped to the genomic regions of *GRIA2*, this gene was not considered to be within the top 10 genes that accounted for age-related methylation changes.

Using the OEAC analysis, we were able to confirm the presence of at least four CpG sites with high correlation to sex as exhibited by the correct identification of the sex in all but two animals (99% accuracy). Both misidentified animals were wild killer whales, one of the samples was from a biopsy collection and one from a dead stranded animal. Both samples passed quality control checks indicating that DNA integrity had not been compromised either from postmortem autolysis in the stranded animal or the sample was too small to provide enough DNA. Despite these misidentified animals, our results provide a case study on the utility of using OEAC analysis for the identification of sex in odontocetes and provide an additional tool to help evaluate the overall health and sustainability of wild populations.

The application of the clock to a wild odontocete population was illustrated by determining the age and sex of 22 free-ranging, Norwegian killer whales, and the results were fairly consistent with previous age estimations of individuals based on field observations^71^. The ability to determine the age and sex of these whales and the continued application of this technique toward a larger representation of the Norwegian killer whale population will provide a more accurate assessment of its demographics to inform potential needs for monitoring and management^72^. Although the overall sample set was small, and significantly biased toward males for unknown reasons, it nonetheless still allowed for a couple of observations. Sex distribution withstanding, the age distribution is similar to that described by Christensen^73^, with some notable differences, including that they found ~5% of the animals were over 30, with the oldest at age of 35 years^73^. We identified 22% of killer whales over age 30, and 5% (two males out of 40 individuals) over age 40 years. However, similar to results from Christenson^73^ and Best et al.^13^ for South African killer whales, no females over age 40 were identified. This contrasts against previous results for southern Alaskan resident killer whales^3^ and northern resident killer whale populations (NRKW) off British Columbia^3^, whereby ~3.3 and 7.7% of the total population were females were over age 40, respectively. While the expected number of females over age 40 was significantly less than what would be expected based on published NRKW demographics, the biased sampling toward males indicates a violation of the random sampling required for this analysis. Therefore, additional sampling would be required before any conclusions about potential populations differences could be made. Future efforts at applying our OEAC or other specific killer whale epigenetic clocks for use in multiple free-ranging killer whale populations would help answer these questions.

Our results provide an advancement in the evidence-based science of age assessment of wild odontocete populations through the application of our OEAC toward either blood or skin samples as described herein. As determined by epigenome-wide association analysis, the highly conserved DNA methylation patterns of age associated CpGs across odontocetes allowed us to produce these extremely accurate clocks, as proven using cross validation techniques, which are now available to aid in age determination of wild toothed whales and dolphins across the Odontoceti suborder.

## Methods

### Ethics approval

The study was authorized by the management of each institution and was reviewed by their respective zoo research and animal use committees.

### Study animals

For model development, our study population included 293 animals from nine species of odontocetes, of which the majority were from four species including beluga (*n* = 66), Pacific white-sided dolphins (*n* = 17), killer whales (*n* = 37), and bottlenose dolphins (*n* = 137), housed at nine Association of Zoos and Aquarium (AZA), Alliance for Marine Mammal Parks and Aquariums or Japanese AZA accredited zoological institutions (Table 1). Known (77.8%) or estimated (based on length at capture or rescue for stranded animals) birth dates were provided by each housing institution. In addition to zoo-based animals, we included 19 skin samples from free-ranging Norwegian killer whales to the training set because the ages of these animals could be estimated within sufficient accuracy (expected error less than 8%) on the basis of several lines of evidence including GLG counts (*n* = 3), whereby 1 GLG was assigned per year of age [12], length at necropsy (*n* = 1), juvenile at first identification (*n* = 2) or minimum age estimation based on length and maturity at first sighting (*n* = 13^72,73^). The remaining 26 animals’ ages could not be estimated within our accuracy parameters and skin samples from these animals were used to demonstrate model application for determining age and sex in wild animals.

### Sample collection

Blood samples (0.5 ml min) were collected either voluntarily from the peripheral periarterial venous rete on the ventral tail fluke using an 18–22 gauge winged blood collection set or attached to a vacutainer collection system. Blood was collected by either the veterinary technician or veterinarian on staff and into BD Vacutainers (Becton Dickinson, Franklin Lakes, NJ) containing EDTA. Samples were inverted in the Vacutainer a minimum of 10 times and then frozen at −80 °C until further testing.

Skin samples (~0.5 gm) were collected either under stimulus control or manual restraint using a sterile disposable dermal curette (Miltex, Integra Life Sciences Corp.,York, PA) from a location just posterolateral of the dorsal fin overlying the epaxial muscle. Prior to collection, a cold pack was placed on the site for several minutes prior to sampling to numb the sample site. Skin samples were placed into sterile cryovials (Nunc® Cryotubes, MilliporeSigma Corp., St. Louis, MO) and stored at −80 °C until shipment on dry ice. Skin samples from non-living animals were obtained from frozen (−80 °C) specimens that had been previously collected and stored during standard necropsy procedures.

Skin samples were sectioned from previously collected killer whale biopsy samples of 45 unique individuals (photo-identified) collected in August and November 2017 and from April through July 2018 in northern Norway^74^. The killer whales were biopsied using an ARTS darting system (Restech, Bodø, Norway) and 25 × 9 mm or 40 × 9 mm stainless steel tips in 2017, and with an injection gun (Pneu-Dart Inc., Williamsport, PA) and 25 × 7 mm tips in 2018 as previously described^74^. In addition, tissue samples were collected from six dead, stranded killer whales, and one other individual by-caught in a herring purse-seine, in northern Norway between 2015 and 2017. Skin samples were collected from the region directly posterior to the dorsal fin and stored at −20 °C until analysis.

### DNA extraction

Genomic DNA was extracted from clotted whole blood samples using QIAamp DNA Mini blood kit and following the manufacturer’s instructions (Qiagen, Valenica, CA). Tissue samples were pulverized and broken down manually using a drill and DNA was extracted using DNeasy Tissue kit (Qiagen) and following the manufacturer’s instructions with the exception of extending the proteinase k digestion. DNA was then extracted using the automated nucleic acid extraction platform, Anaprep (Biochain, Newark, CA) that utilizes a magnetic bead extraction process and Tissue DNA Extraction kit (Anaprep).

### DNA methylation data

We generated DNA methylation data using the custom Illumina chip (HorvathMammalMethylChip40^75^). The mammalian methylation array provides high coverage (over thousand-fold) of highly conserved CpGs in mammals but focuses only on 36k CpGs that are highly conserved across mammals. Out of 37,492 CpGs on the array, 35,988 probes were chosen to assess cytosine DNA methylation levels in mammalian species^75^. The particular subset of species for each probe is provided in the chip manifest file can be found at Gene Expression Omnibus (GEO) at NCBI as platform GPL28271. The SeSaMe normalization method was used to define beta values for each probe^76^.

### Clocks and penalized regression

Penalized regression models were implemented with the R software package, glmnet^77^. We investigated models produced using “LASSO” regression techniques (*α* = 1). The optimal penalty parameters in all cases were determined automatically by using a 10-fold internal cross-validation (cv.glmnet) on the training set. *α* = 1/2 corresponds to the elastic net penalty that penalizes the coefficients based on their magnitude. We performed two cross-validation schemes for arriving at unbiased estimates of the accuracy of the different DNAm aging clocks. These cross-validation schemes were as follows: (1) Leave One Sample Out Cross Validation (LOOCV) which is based on previously reported methods;^78,79^ and (2) Leave One Species Out Cross-Validation (LOSOCV), which is a modification of LOOCV that is applied to species instead of individual samples as described in the following. The LOOCV does the following for each of the *N* samples: delete the one sample from the training set; fit the clock on the training set with (*N*−1) samples; predict the DNAm age of the deleted sample with the fitted clock. Therefore, the LOOCV allows one to estimate the accuracy of each individual from all species that were included in the training set. By contrast, the LOSOCV iteratively deletes all samples from one species from the training set and then predicts the age of each individual of the species that was removed from the training set. This procedure estimates the accuracy of the OEAC at determining the age of an animal from an odontocete species that was not used for model development. The cross validation study reports unbiased estimates of the age correlation *r* (defined as Pearson correlation between the DNAm age estimate and chronological age) as well as the median absolute error (MAE). For the odontocete clock, we used a log-linear transformation that explicitly depended on the species characteristics (average age of sexual maturity and gestation time (in years) of the respective species, Supplementary Methods, Supplementary Table 1). The accuracy of the resulting clocks was assessed via the cross validation estimates of: (1) the correlation *r* between the predicted epigenetic age and the actual (chronological) age of the animal; and (2) the median absolute error (MAE) between DNAm age and chronological age.

### Statistics and reproducibility

Data collection, and statistical analysis are described in Methods section. All statistical analysis for clock development used R Software (ver 4.0.2)^80^. Relevant R codes for the clock development are provided (Supplementary Data File 2). Penalized regression models were implemented with the R software package “glmnet”^77^ and R functions cv.glmnet, predict.glmnet, etc. Data transformations used within regression models are described in Supplementary Information File (Supplementary Methods 1 and Supplementary Table 1).

### Reporting summary

Further information on research design is available in the Nature Research Reporting Summary linked to this article.

## Supplementary information

Supplementary Information

Description of Additional Supplementary Files

Supplementary Data 1

Supplementary Data 2

Supplementary Data 3

Reporting Summary

## Data Availability

Details on the CpGs (genome coordinates) used for clock development are provided in Supplementary Data File 1. Source data underlying the main figures are available in Supplementary Data File 3. The DNA methylation data underlying this publication be found on Gene Expression Omnibus (GSE173330). Genome annotations of these CpGs can be found on Github: https://github.com/shorvath/MammalianMethylationConsortium.

## Code availability

The R software code used for clock development are provided in the Supplementary Data File 2.
