# Supplementary material for: Multi-species and multi-tissue methylation clocks for age estimation in toothed whales and dolphins
Source: Commun Biol. 2021 May 31;4:642. doi: 10.1038/s42003-021-02179-x (PMC8167141; doi:10.1038/s42003-021-02179-x)
Supplement: Supplementary file 1 — Supplementary Information [file 42003_2021_2179_MOESM1_ESM.pdf]

Supplementary Information for:

Multi-species and multi-tissue methylation clocks for age estimation in toothed whales and dolphins

Todd R. Robeck[1#], Zhe Fei[2#], Ake T. Lu[3], Amin Haghani [3], Eve Jourdain[4], Joseph A. Zoller[2], Caesar Z. Li[2], Karen J. Steinman[5], Stacy DeRocco[6], Todd Schmitt[7], Steve Osborn[8], Bill Van Bonn[9], Etsuko Katsumata[10], June Mergl[11], Javier Almunia[12], Magdalena Rodriguez[13], Martin Haulena[14], Christopher Dold[1], Steve Horvath[2,3*ǂ]

[#]Joint First Authorship
[ǂ]*Corresponding authors*
Steve Horvath, PhD, ScD
E-mail: shorvath@mednet.ucla.edu
Todd Robeck, PhD, DVM
E-mail: todd.robeck@seaworld.com

This PDF file includes

Supplementary Methods
Supplementary Table 1
Supplementary Figures 1 to 6
Supplementary References

Supplementary Methods

Statistical details for epigenetic clock development.

All of the odontocete blood+skin clock and tissue specific clocks use the same age transformation (Supplementary Table 1). Denote sample i from species k has Age X_{ik} and Age at sexual maturity for species k is a_k, Gestation time (in years) for species k is g_k. Then the log-linear transformation is as follows (Equation 1):

$$\text{Equation 1.} \quad Y_{ik} = \begin{cases} \log(\frac{X_{ik} + g_k}{1.5a_k + g_k}), & X_{ik} \leq 1.5a_k \\ \frac{X_{ik} - 1.5a_k}{1.5a_k + g_k}, & X_{ik} > 1.5a_k. \end{cases}$$

Adding gestation time in the transformation means age since inception and avoids negative values in the log. This transformation ensures the transformed age Y_{ik} is continuous and has continuous first derivative at $X_{ik} = 1.5a_k$.

Supplementary Table 1. Mean age (male and female) at sexual maturity and gestation time in years for each respective species used in epigenetic clock development.

Species: common name (scientific name)	Sexual Maturity	Gestation length
bottlenose dolphin (*Tursiops truncatus*)	8.93	1.03
beluga (*Delphinapterus leucas*)	11.0	1.28
Commerson's dolphin (*Cephalorhynchus commersonii*),	5.5	0.96
common dolphin (*Delphinus delphis*)	4.57	0.96
harbor porpoise (*Phocoena phocoena*)	3.45	0.88
killer whale (*Orcinus orca*	10.5	1.46
Pacific white-sided dolphin (*Lagenorhynchus obliquidens*)	7.0	0.98
rough-toothed dolphin (Steno bredanensis).	10.0	1.03
short-finned pilot whales (*Globicephala macrorhynchus*),	12.06	1.24

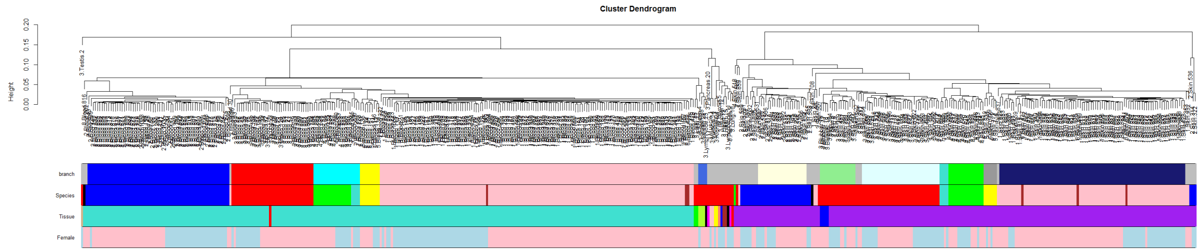

Supplementary Fig. 1 Unsupervised hierarchical clustering of tissue samples. Average linkage hierarchical clustering based on the interarray correlation coefficient (Pearson correlation). The branch colors (first color band) largely correspond to tissue type (third color band) and to a lesser extent on species (second color band). The individual leaves of the tree are labeled by species number (as in Fig 1): 1 = bottlenose dolphin (*Tursiops truncatus*); 2 = beluga (*Delphinapterus leucas*); 3 = killer whale (*Orcinus orca*); 4 = Pacific white-sided dolphin (*Lagenorhynchus obliquidens*); 5 = short-finned pilot whales (*Globicephala macrorhynchus*); 6 = common dolphin (*Delphinus delphis*); 7 = Commerson's dolphin (*Cephalorhynchus commersonii*); 8 = harbor porpoise (*Phocoena phocoena*). Explanation of the first color band: the blue branch corresponds to blood samples from beluga; turquoise branch = blood from Pacific white-sided dolphin; red = blood from killer whales; yellow = blood from short-finned pilot whales; pink = blood from bottlenose dolphin; light cyan/blue = skin from beluga; light green = skin from killer whales; midnight blue = blubber and skin from killer whale; green = skin from Pacific white-sided dolphin; salmon = skin from bottlenose dolphin.

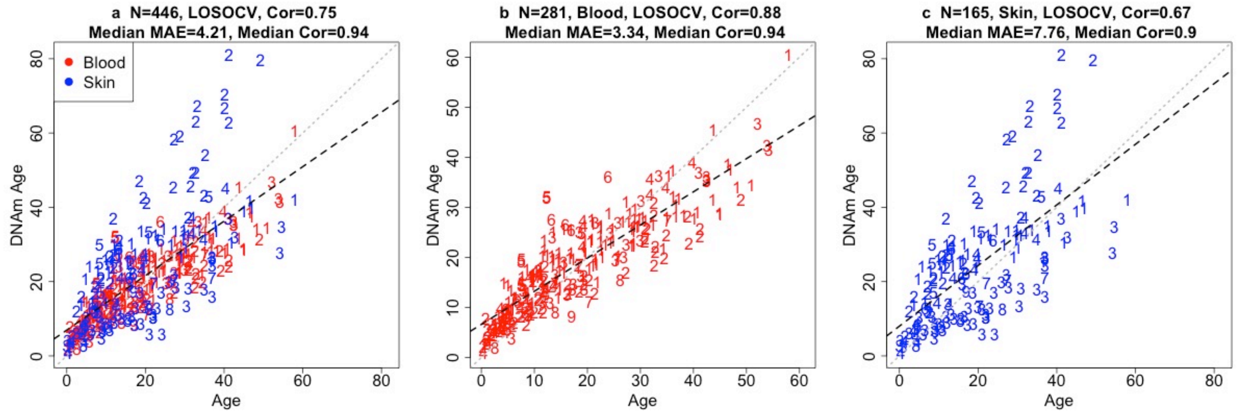

Supplementary Fig. 2. Leave-One-Species Out Cross-validation (LOSOCV) study of the epigenetic clock for odontocetes. Each dot corresponds to a tissue sample from odontocetes. Dots are colored by tissue type (red = blood, blue = skin) and labeled by species: 1 = bottlenose dolphin (*Tursiops truncatus*); 2 = beluga (*Delphinapterus leucas*); 3 = killer whale (*Orcinus orca*); 4 = Pacific white-sided dolphin (*Lagenorhynchus obliquidens*); 5 = short-finned pilot whales (*Globicephala macrorhynchus*); 6 = rough-toothed dolphin (*Steno bredanensis*); 7 = Commerson's dolphin (*Cephalorhynchus commersonii*); 8 = common dolphin (*Delphinus delphis*); 9 = harbor porpoise (*Phocoena phocoena*). Panels (a - c) represent cross validations of different tissue strata, with blood and skin combined (a), blood only (b) and skin only (c). The LOSOCV estimates lend themselves for estimating the performance in any odontocete species that were not part of the training set. Each panel depicts a linear regression line (black dashed line), a diagonal line (y = x), the sample size (N), Pearson correlation (Cor) across all samples, median age correlation across species, median value of the median absolute error (MAE) across species.

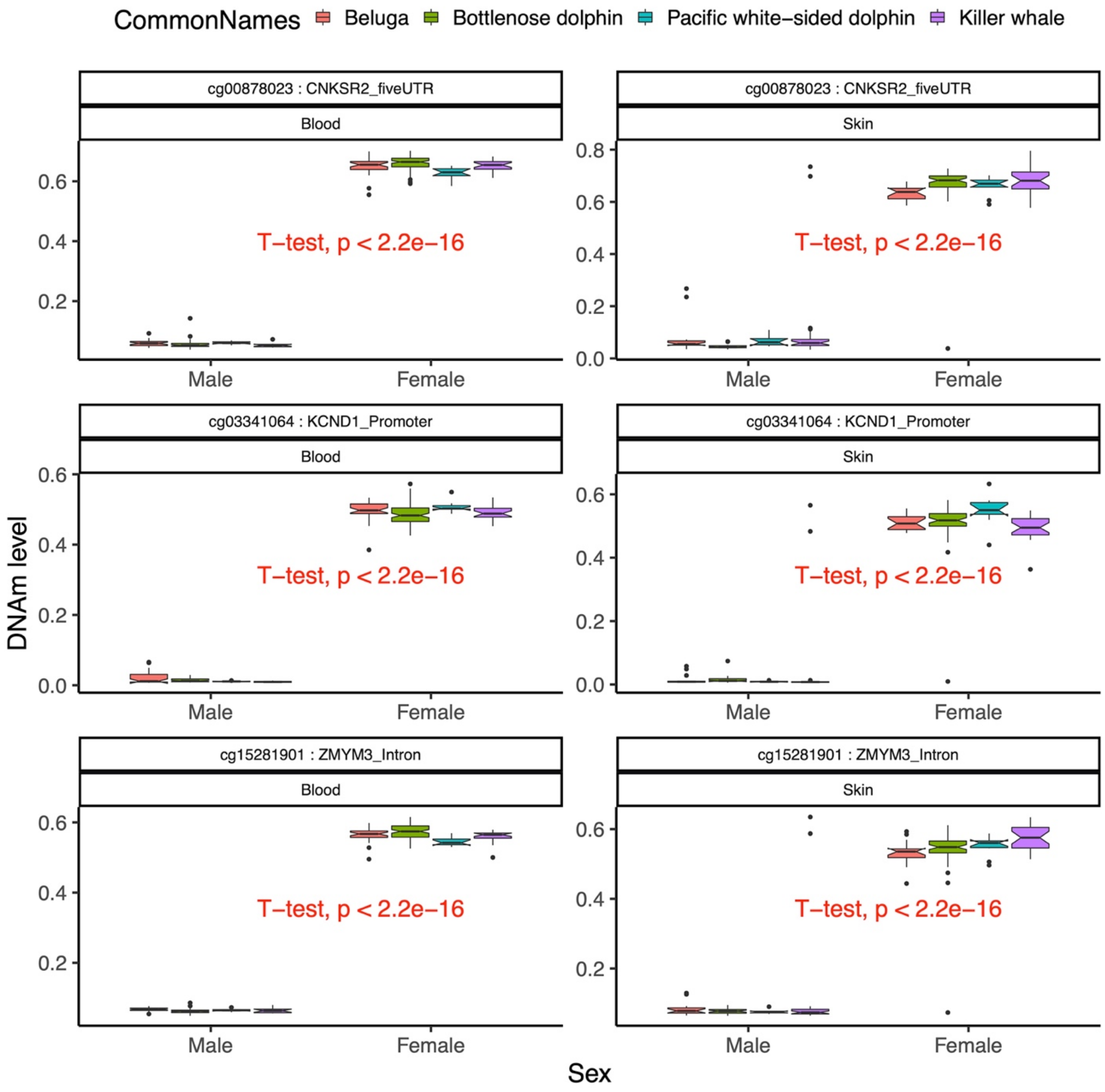

Supplementary Fig. 3. **Select CpGs across all species that were highly associated with sex across odontocetes.** The box plots represent the sex differences in all available samples across all ages. The reported t.test is the mean methylation differences between sexes across all odontocetes.

\

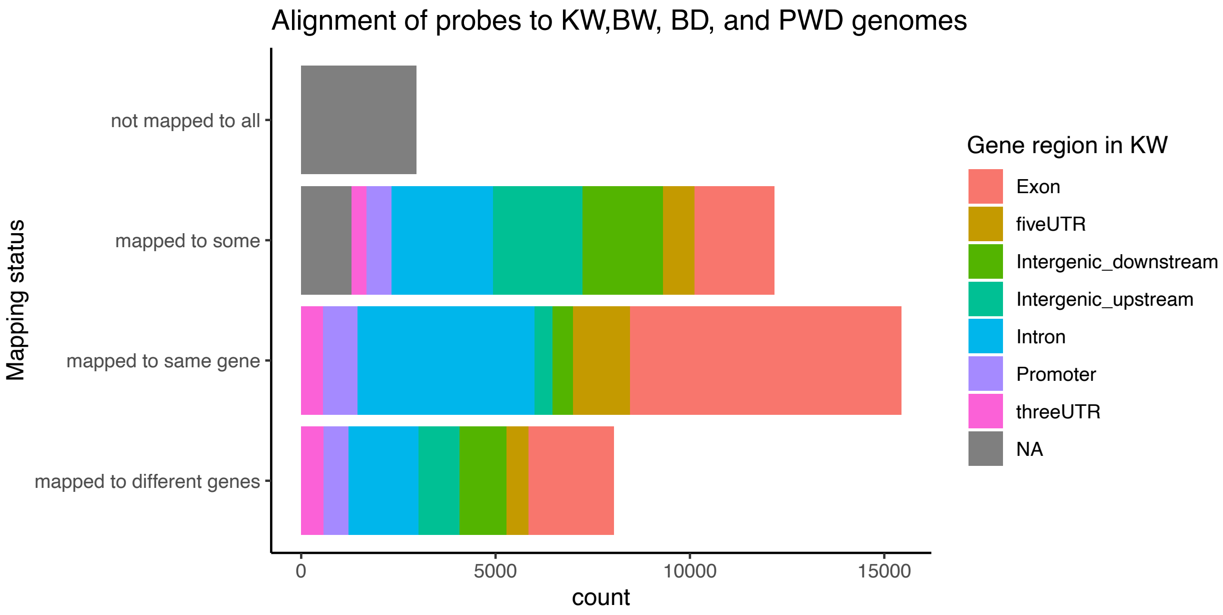

Supplementary Fig. 4. Comparison of mammalian array coverage in killer whale (KW), beluga whale (Beluga), bottlenose dolphin (BD), and Pacific white-sided dolphin (PWD) genomes. Genome assemblies: KW.GCF_000331955.2_Oorc_1.1; Beluga.ASM228892v3.100; Tursiops_truncatus.turTru1.100; Lagenorhynchus_obliquidens.ASM367639v1. Categories: mapped to same gene in all four species; mapped to some, which means it mapped at least to one of these species; mapped to the same gene in all four species; mapped to different genes.

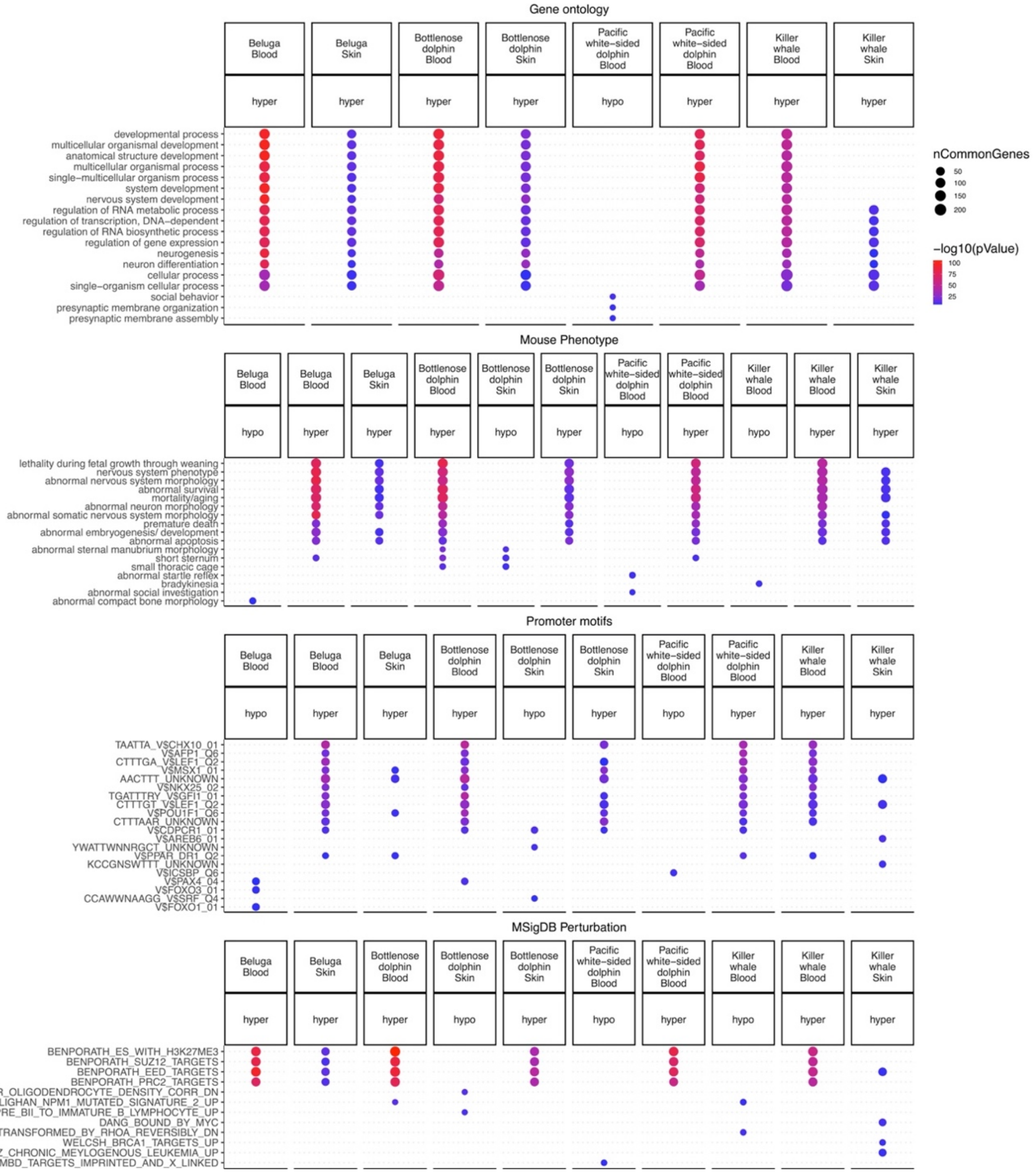

Supplementary Fig. 5. Enrichment analysis of the top DNAm aging marks in odontocete tissues. Gene set enrichment analysis of proximate genes with DNAm aging in odontocete tissues. The gene level enrichment was done using the genomic region of enrichment annotation tool (GREAT, McLean et al., 2010) and human Hg19 background (McLean et al., 2010). The top three enriched datasets from each category (Gene ontology, mouse phenotypes, Promoter motifs, and MSigDB Perturbation) were selected and further filtered for significance at $P < 10^{-7}$.

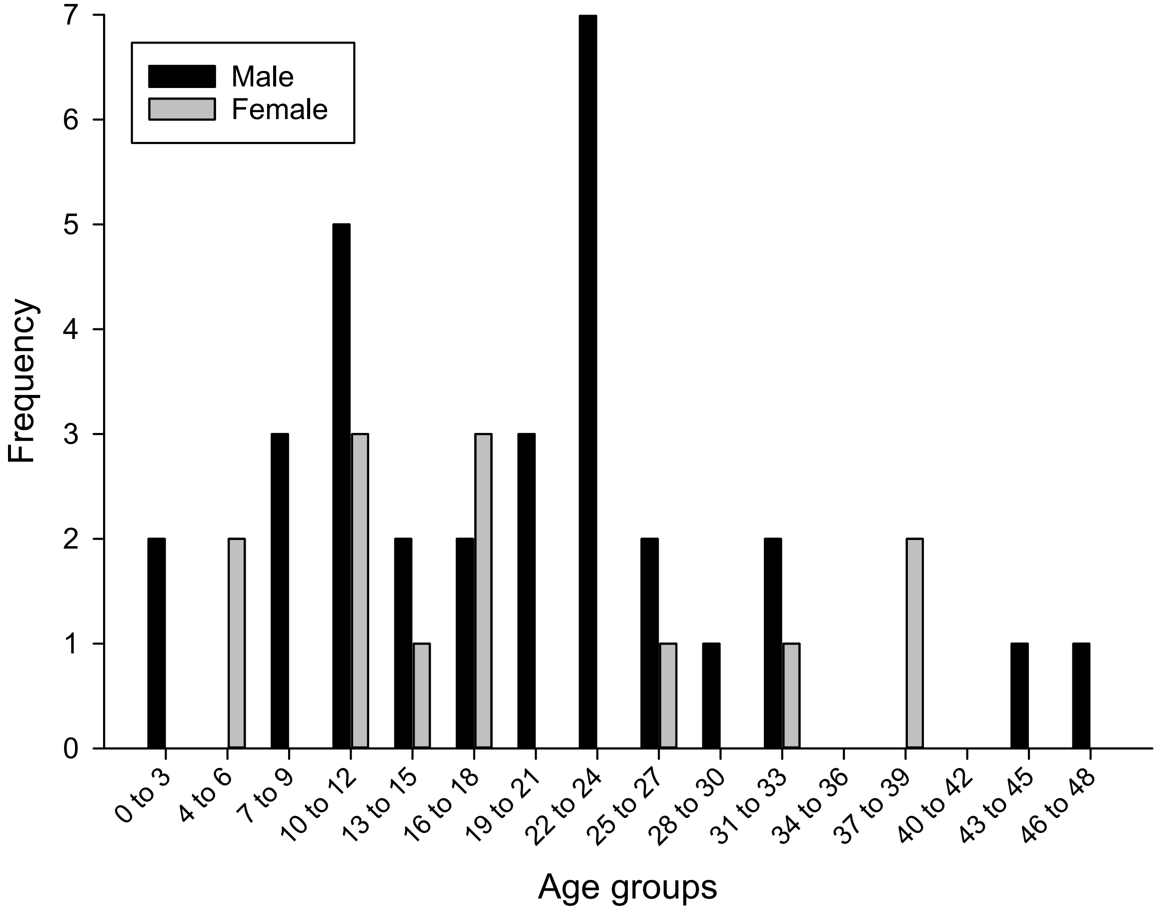

Supplementary Fig. 6. Population demographics (age and sex) of Norwegian killer whales used within this research. Age was determined by either direct application of the Odontocete Epigenetic Aging Clock blood skin clock (OEAC) to predict age of animals from skin samples (n = 21) or known age animals (n = 19). Known age animal ages were highly correlated with OEAC predicted ages (r = 0.92).

Supplementary References.

1. C. Y. McLean, *et al.*, GREAT improves functional interpretation of cis-regulatory regions. *Nat. Biotechnol.* **28**, 495-501 (2010). doi: 10.1038/nbt.1630.
