# Supplementary material for: Multi-species and multi-tissue methylation clocks for age estimation in toothed whales and dolphins
Source: Commun Biol. 2021 May 31;4:642. doi: 10.1038/s42003-021-02179-x (PMC8167141; doi:10.1038/s42003-021-02179-x)
Supplement: Supplementary file 2 — Description of Additional Supplementary Files [file 42003_2021_2179_MOESM2_ESM.pdf]

Description of Additional Supplementary Files

File name: Supplementary Data File 1.

Description: *CpGs used for clock development*. There are three clocks, found on individual worksheets within the spreadsheet, being developed with the proposed method and using R glmnet function, Blood+Skin, Blood and Skin. Take the first clock, Odontocete Blood+Skin clock for example, 142 CpGs were selected by `cv.glmnet` with an intercept term, whose coefficients are non-zero. Readers can use the clock on their methylation data for prediction by, 1. Calculate the linear predictor of methylation vector times CpG coefficient vector; 2. Perform the inverse log-linear transformation as described in Supplementary Methods to obtain the age prediction.

File name: Supplementary Data File 2.

Description: *R Source Code for epigenetic clock development*. In the R code, we specified the data, the age transformation (function `logli`) and its inverse function and the different age estimates: Training estimates, LOO estimates, and LOSO estimates, as well as the selected CpGs for each of the final three clocks.

File name: Supplementary Data File 3.

Description: The underlying source data for Fig. 1, Fig. 2., Fig 3., and Fig. 4 found on individual worksheets within the excel file.
