# Supplementary material for: Multi-species and multi-tissue methylation clocks for age estimation in toothed whales and dolphins
Source: Commun Biol. 2021 May 31;4:642. doi: 10.1038/s42003-021-02179-x (PMC8167141; doi:10.1038/s42003-021-02179-x)
Supplement: Supplementary file 4 — Supplementary Data 2 [file 42003_2021_2179_MOESM4_ESM.docx]

**Supplementary Information for:**

**Multi-species and multi-tissue methylation clocks for age estimation in toothed whales and dolphins**

Todd R. Robeck and Zhe Fei et al..

**This file includes:**

**Supplementary Data File 2. R Source Code for epigenetic clock development**.

library(colorspace)

require(RNOmni)

require(glmnet)

require(caret)

library(plyr)

library(RColorBrewer)

library(WGCNA)

library(tidyverse)

outfolder = "OdoClocks"

load("../all_probes_all_samples_sesame.RData")

if (!is.numeric(dat0sesame[,1])) {

rownames(dat0sesame) <- dat0sesame[,1]

dat0sesame <- dat0sesame[,-1]

}

id1 = substr(rownames(dat0sesame),1,2) != "rs"

table(id1)

dat0sesame = dat0sesame[id1,]

dat1 = read.csv("../SampleSheetAgeN27final_SamplesForOdoPaper.csv",row.names = 1)

dat1$CetaceanNumber <- NA

kk = k = length(tabN27)

while (k>0) {

spec.name <- names(tabN27)[k]

print(c(k, spec.name))

idx1 = which(dat1$SpeciesLatinName == spec.name)

dat1$Size[idx1] <- length(idx1)

dat1$CetaceanNumber[idx1] <- kk+1-k

k=k-1

}

table(dat1$CetaceanNumber)

dat1$tissueColor[dat1$Tissue=="Blood"] <- "red"

dat1$tissueColor[dat1$Tissue=="Skin"] <- "blue"

dat1$SpeciesCommonName[dat1$SpeciesCommonName == "Beluga whale"]<- "Beluga"

dat1$SpeciesCommonName[dat1$SpeciesCommonName == "Pacific white-sided dolphin"]<- "Pacific ws dolphin"

#############################

#

######## Modeling

#

#############################

################ Age transformation

logli<- function(age1, m1, m2 = m1, c1=1){

ifelse(age1 >= m1, (age1-m1)/m2 , c1*log((age1-m1)/m2/c1 +1) )

}

rev_logli = function(y.pred, m1, m2=m1, c1=1){

ifelse(y.pred<0, (exp(y.pred/c1)-1)*m2*c1 + m1, y.pred*m2+m1 )

}

trsf = logli

revtrsf = rev_logli

dim(dat1)

m1 = 1.5

x = dat1$Age + dat1$Gestation ### 1

c1m = m1*dat1$AvgMaturity + dat1$Gestation

y = logli(x, c1m)

hist(y)

dat1$tAge <- y

summary(dat1$tAge)

tabN27 <- sort(table(dat1$SpeciesLatinName))

cg.idx = 1:nrow(dat0sesame)

############### training all samples

train.idx <- pmatch(dat1$Basename, colnames(dat0sesame))

y.train = dat1$tAge

t1=proc.time()

fit1 <- cv.glmnet(t(dat0sesame[cg.idx,train.idx]), y.train, family="gaussian",alpha = 1/2) ###, weights = weights)

#print(proc.time() - t1)

plot(fit1)

y.fitted <- predict(fit1,t(dat0sesame[cg.idx,train.idx]), s="lambda.1se")

verboseScatterplot(dat1$tAge, y.fitted)

summary(y.fitted)

dat1$DNAmYOdoClockTraining = y.fitted

age.fitted <- revtrsf(y.fitted, c1m) - dat1$Gestation

verboseScatterplot(dat1$Age, age.fitted)

dat1$DNAmAgeOdoClockTraining <- NA

dat1$DNAmAgeOdoClockTraining <- age.fitted

lam1se = fit1$lambda.1se

odoClock = fit1

coef1 = coef(fit1, s="lambda.1se")

sum(coef1!=0)

coefmat <- data.frame("Cg"=rownames(coef1)[which(coef1!=0)],"Coef"= as.numeric(coef1[coef1!=0]))

head(coefmat)

write.csv(coefmat,paste0(outfolder,"/OdoClock_",Sys.Date(), ".csv"))

########## LOO

lam1se = fit1$lambda.1se

dat1$DNAmYOdoClockLOO <- NA

for(i in 1:nrow(dat1)){

train.idx <- pmatch(dat1$Basename[-i], colnames(dat0sesame))

y.train = dat1$tAge[-i]

test.idx = pmatch(dat1$Basename[i], colnames(dat0sesame))

fit1 <- glmnet(t(dat0sesame[cg.idx,train.idx]), y.train,lambda = lam1se,

family="gaussian",alpha = 1/2) ###, weights = weights)

y.fitted <- predict(fit1,t(dat0sesame[cg.idx,test.idx]))

dat1$DNAmYOdoClockLOO[i] <- y.fitted

print(i)

}

summary(dat1$DNAmYOdoClockLOO)

y = dat1$DNAmYOdoClockLOO

verboseScatterplot(dat1$tAge, y)

abline(0,1)

age.fitted <- revtrsf(y, c1m) - dat1$Gestation

verboseScatterplot(dat1$Age, age.fitted, type="n")

text(dat1$Age, age.fitted, dat1$MammalNumberHorvath)

abline(0,1)

cor(dat1$Age, age.fitted)

dat1$DNAmAgeOdoClockLOO <- NA

dat1$DNAmAgeOdoClockLOO <- age.fitted

tabN27 <- sort(table(dat1$SpeciesLatinName))

tabN27

######## Odo blood and skin clocks

length(cg.idx)

for(tis in c("Blood","Skin")){

tis

idx1 = which(dat1$Tissue == tis)

train.idx <- pmatch(dat1$Basename[idx1], colnames(dat0sesame))

y.train = dat1$tAge[idx1]

t1=proc.time()

fit1 <- cv.glmnet(t(dat0sesame[cg.idx,train.idx]), y.train, family="gaussian",alpha = 1/2) ###, weights = weights)

#print(proc.time() - t1)

plot(fit1)

coef1 = coef(fit1, s="lambda.1se")

sum(coef1!=0)

coefmat <- data.frame("Cg"=rownames(coef1)[which(coef1!=0)],"Coef"= as.numeric(coef1[coef1!=0]))

head(coefmat)

write.csv(coefmat,paste0(outfolder,"/Odo",tis, "Clock_",Sys.Date(), ".csv"))

lam1se = fit1$lambda.1se

dat1$DNAmY <- NA

for(i in 1:length(idx1)){

tmpi = idx1[i]

train.idx <- pmatch(dat1$Basename[idx1[-i]], colnames(dat0sesame))

y.train = dat1$tAge[idx1[-i]]

test.idx = pmatch(dat1$Basename[tmpi], colnames(dat0sesame))

fit1 <- glmnet(t(dat0sesame[cg.idx,train.idx]), y.train,lambda = lam1se,

family="gaussian",alpha = 1/2) ###, weights = weights)

y.fitted <- predict(fit1,t(dat0sesame[cg.idx,test.idx]))

dat1$DNAmY[tmpi] <- y.fitted

print(i)

}

summary(dat1$DNAmY)

y = dat1$DNAmY

verboseScatterplot(dat1$tAge, y)

abline(0,1)

# c1m=m1*dat1$AvgMaturity

age.fitted <- revtrsf(y, c1m) - dat1$Gestation

summary(age.fitted)

xr = range(c(dat1$Age[idx1], age.fitted[idx1]), na.rm = T)

verboseScatterplot(dat1$Age[idx1], age.fitted[idx1], type="n",xlim=xr,ylim=xr)

text(dat1$Age[idx1], age.fitted[idx1], dat1$MammalNumberHorvath[idx1])

abline(0,1)

dat1$DNAmAge <- NA

dat1$DNAmAge <- age.fitted

nc = ncol(dat1)

tmp1 = colnames(dat1)[c(nc-1,nc)]

colnames(dat1)[c(nc-1,nc)] = paste0(tmp1,"Odo",tis,"ClockLOO")

}

summary(dat1)

########## LOSO

tabN27 <- sort(table(dat1$SpeciesLatinName))

tabN27

dat1$DNAmYOdoClockLOSO <- NA

for(i in 1:length(tabN27)){

specname = names(tabN27)[i]

idx1 = which(dat1$SpeciesLatinName == specname)

train.idx <- pmatch(dat1$Basename[-idx1], colnames(dat0sesame))

y.train = dat1$tAge[-idx1]

test.idx = pmatch(dat1$Basename[idx1], colnames(dat0sesame))

fit1 <- cv.glmnet(t(dat0sesame[cg.idx,train.idx]), y.train,

family="gaussian",alpha = 1/2) ###, weights = weights)

y.fitted <- predict(fit1,t(dat0sesame[cg.idx,test.idx]), s="lambda.1se")

dat1$DNAmYOdoClockLOSO[idx1] <- y.fitted

print(i)

}

summary(dat1$DNAmYOdoClockLOSO)

y = dat1$DNAmYOdoClockLOSO

verboseScatterplot(dat1$tAge, y)

abline(0,1)

m1

age.fitted <- revtrsf(y, c1m) - dat1$Gestation

verboseScatterplot(dat1$Age, age.fitted, type="n")

text(dat1$Age, age.fitted, dat1$MammalNumberHorvath)

abline(0,1)

dat1$DNAmAgeOdoClockLOSO <- NA

dat1$DNAmAgeOdoClockLOSO <- age.fitted

colnames(dat1)

write.csv(dat1,paste0(outfolder,"/datSampOdoPaper.csv"))

######### Figures

source("CodeForFigures.R")
